# BSMM-Net: Multi-modal neural network based on bilateral symmetry for nasopharyngeal carcinoma segmentation

**DOI:** 10.3389/fnhum.2022.1068713

**Published:** 2023-01-10

**Authors:** Haoyang Zhou, Haojiang Li, Shuchao Chen, Shixin Yang, Guangying Ruan, Lizhi Liu, Hongbo Chen

**Affiliations:** ^1^School of Life & Environmental Science, Guangxi Colleges and Universities Key Laboratory of Biomedical Sensors and Intelligent Instruments, Guilin University of Electronic Technology, Guilin, Guangxi, China; ^2^State Key Laboratory of Oncology in South China, Collaborative Innovation Center for Cancer Medicine, Sun Yat-sen University Cancer Center (SYSUCC), Guanghzou, Guangdong, China; ^3^School of Electronic Information and Automation, Guilin University of Aerospace Technology, Guilin, Guangxi, China

**Keywords:** segmentation, MRI, neural network, multi-modal, nasopharyngeal carcinoma

## Abstract

**Introduction:**

Automatically and accurately delineating the primary nasopharyngeal carcinoma (NPC) tumors in head magnetic resonance imaging (MRI) images is crucial for patient staging and radiotherapy. Inspired by the bilateral symmetry of head and complementary information of different modalities, a multi-modal neural network named BSMM-Net is proposed for NPC segmentation.

**Methods:**

First, a bilaterally symmetrical patch block (BSP) is used to crop the image and the bilaterally flipped image into patches. BSP can improve the precision of locating NPC lesions and is a simulation of radiologist locating the tumors with the bilateral difference of head in clinical practice. Second, modality-specific and multi-modal fusion features (MSMFFs) are extracted by the proposed MSMFF encoder to fully utilize the complementary information of T1- and T2-weighted MRI. The MSMFFs are then fed into the base decoder to aggregate representative features and precisely delineate the NPC. MSMFF is the output of MSMFF encoder blocks, which consist of six modality-specific networks and one multi-modal fusion network. Except T1 and T2, the other four modalities are generated from T1 and T2 by the BSP and DT modal generate block. Third, the MSMFF decoder with similar structure to the MSMFF encoder is deployed to supervise the encoder during training and assure the validity of the MSMFF from the encoder. Finally, experiments are conducted on the dataset of 7633 samples collected from 745 patients.

**Results and discussion:**

The global DICE, precision, recall and IoU of the testing set are 0.82, 0.82, 0.86, and 0.72, respectively. The results show that the proposed model is better than the other state-of-the-art methods for NPC segmentation. In clinical diagnosis, the BSMM-Net can give precise delineation of NPC, which can be used to schedule the radiotherapy.

## 1. Introduction

Nasopharyngeal carcinoma (NPC) is a common malignant tumor with geographical distribution, mainly in Southeast Asia, North Africa, and the Mideast (Tatanun et al., 2010). According to the American Joint Committee on Cancer staging system, clinicians and radiologists can stage patients according to the laterality, quantity, size, and location of the primary NPC tumors. Head magnetic resonance imaging (MRI) is the first choice for diagnosing NPC and delineating primary NPC tumors because T1-weighted MRI (T1) can reflect the skull base involvements and T2-weighted MRI (T2) can provide better structural information on soft tissues than T1. However, exactly outlining the primary NPC tumors from T1 and T2 head MRI images for staging is tiring and time-consuming. Therefore, developing an accurate and efficient NPC segmentation method is crucial to assist radiologists.

According to the types of features, the methods for accurate primary NPC tumor segmentation can be roughly grouped into two major classes, namely, traditionally handcrafted feature-based and deep feature-based. Traditionally handcrafted feature-based works utilize manual features, such as intensity, texture, and shape, to generate the NPC boundary (Zhou et al., 2006; Tatanun et al., 2010; Chanapai et al., 2012; Huang et al., 2013, 2015). However, accurately contouring the NPC boundary on the basis of these simply handcrafted features is difficult because of the fuzzy boundary and variable location of NPC lesions.

Previous studies have achieved excellent improvement using deep features extracted by deep neural networks and linked with the segmenting task in training. Some deep feature-based works improve accuracy by utilizing multi-level and multi-scale information of deep features (Li et al., 2018; Ke et al., 2020). However, these works ignore the spatial relationships of the head and complementary information of different modalities. Although some researchers utilized the spatial relationships through variant transformers, the large NPC dataset needed to train the fully connected layers of transformers is difficult to collect (Khan et al., 2021; Dhamija et al., 2022). Although these models can be properly trained, the possibility of learning the bilateral symmetry of the head is unclear and the spatial relationship of the multi-head attention mechanism is not explainable. T-staging information has been transferred into a channel, stacked with other modalities, and fed into an attention Unet to segment the NPC tumors (Cai et al., 2021). However, achieving the T-staging information is difficult and time-consuming. Semi-supervised models are hot spots for less number of NPC datasets, but the performance of these models still cannot be compared to the models trained by all labeled datasets (Luo et al., 2021; Hu et al., 2022). Some studies focused on variable locations and irregular boundaries of NPC tumors but paid less attention to the bilateral symmetry of the head and complementary information of multiple modality-specific features (Li et al., 2022).

To address these issues, this research proposes a deep feature-based network that can detect NPC tumors on the basis of the bilateral symmetry of the head and can fully utilize the complementary information of T1 and T2 modalities. First, the difference value between T2 and T1 (DT) is treated as an individual modality for utilizing the information of high-water-content tissues, such as the cerebellum and spinal cord. Bilaterally symmetrical patch block (BSP) directly crops the image and its horizontally flipped image at the same location with the same size to precisely locate NPC tumors using the information on the bilateral symmetry of the head. The modality-specific and multi-modal fusion feature (MSMFF) encoder is designed to extract deep features containing the complementary information of T1 and T2 from the patches preprocessed by BSP and DT. In addition, the MSMFF encoder blocks are composed of six modality-specific networks and one multi-modal fusion network in parallel. A spatial attention mechanism is then introduced into the base decoder to enhance the features of lesions and obtain accurate segmentation. Finally, the MSMFF decoder with a similar structure to the MSMFF encoder is deployed to deeply supervise the encoder during training and ensure its validity of the MSMFF encoder. The proposed model can precisely locate and delineate NPC tumors on the basis of the bilateral symmetry of the head and the complementary information of T1 and T2 modalities. The main contributions of this study are summarized as follows:

(a) This study is a novel attempt to validate the feasibility of locating NPC lesions on the basis of the bilateral symmetry of the head. In addition, it is a simulation that the radiologist could detect NPC tumors by comparing the difference between the left and right sides of the head.
(b) In order to fully utilize the complementary information and bilateral symmetry of modalities, the MSMFF encoder is proposed to extract the MSMFFs from the patches of T1, T2, DT, and their bilaterally flipped ones. Among these modalities, the DT modality which is computed by subtracting T1 from T2 can enhance the structural and positional information of high-water-content materials such as the cerebellum and spinal cord. The proposed MSMFF encoder is composed of six modality-specific networks and a multi-modal fusion network in parallel. To assure the validity of the MSMFF encoder features, an MSMFF decoder is applied as an auxiliary decoder to deeply supervise the MSMFF encoder during training.
(c) Experiments on the MRI of 745 patients demonstrate that the proposed method obtains improved results by combining the bilateral symmetry of the head and complementary information of T1 and T2 modalities compared with state-of-the-art techniques.

## 2. Related studies

Deep learning networks have excellent feature learning ability and have been widely deployed to combine multi-modality images (such as CT, T1 MRI, and T2 MRI) for organ segmentation or lesion segmentation. Some studies utilized deep learning networks to automatically delineate NPC tumors. Men et al. (2017) extracted the deep features of CT images through a VGG-16-like encoder and utilized a decoder to recover the original resolution. SI-Net, a variant of Unet, showed an improved performance by utilizing the information of the adjacent image and the high-risk primary tumor contour of the adjacent image (Xue et al., 2020). To automatically delineate NPC lesions in MRI images, Wong et al. (2021) used the texture and position information to weigh the channels of skipping features in an Unet-like network. Bai et al. (2021) achieved the rough segmentation of NPC lesions in CT images using ResNeXt-50 Unet, which is constructed by replacing the encoder of Unet with ResNeXt-50. A series of patches sampled based on the rough delineation were then fed into another ResNeXt-50 Unet model to predict the NPC tumor region. Finally, these predictions were merged into the final segmentation. Guo et al. (2020) utilized low-level features and multi-scale information to improve delineation by applying long-range skip connection and multi-scale feature pyramid. For multi-scale and multi-level information, DDNet introduces dense connections and feature pyramids to the networks and achieves excellent performance in NPC MRI (Li et al., 2021). DA-DSUnet exhibits an improved performance by utilizing multi-level features based on channel attention and position attention mechanisms (Tang et al., 2021).

Although the abovementioned methods utilize multi-scale, multi-level, adjacent, positional, or texture information in various ways to enhance the performance, the complementary information of different modalities and the bilateral symmetry of the head, which are a concern of radiologists in contouring NPC tumors, are missed. To improve segmentation performance, Lin et al. (2019) concatenated four modalities and fed the concatenation into a network with residual mechanism and long-range skip connection. Zhao et al. (2019) fed dual-modality PET-CT images into an Unet-like network with auxiliary paths to introduce deep supervision and allow the hidden layers of the decoder to learn additional representative features. For precisely contouring NPC tumors and lymph nodes, NPCNet utilizes ResNet-101 to extract features and then enhances these features by channel attention, spatial attention, and object contextual representation block (Li et al., 2022).

However, the aforementioned multi-modal networks simply fused modalities at the first layer by directly feeding the stacking modalities. Additionally, the means of these networks that are designed for achieving better performance can also be utilized in single-modality tasks. Modality-specific networks that can aggregate complementary information from different modalities are valuable for multi-modal networks (Zhu et al., 2016; Lan et al., 2022). MMFNet uses three specific encoders to separately extract modality-specific features from corresponding modality images. The modality-specific features are fused by a 3D spatial attention module before being fed into the decoders (Chen et al., 2020). Zhu et al. (2022) extracted the modality-specific features of NPC multi-modal images such as MMFNet and fused them with a channel attention module (CAM) to achieve complementary information.

Although some of the abovementioned works utilized modality-specific features to extract additional representative features, the discarded low-level multi-modal fusion features are also crucial in aggregating the complementary information of modalities (Lan et al., 2022). To solve this problem, a multi-modal fusion network is deployed in MSMFF encoder (or decoder) blocks to fuse the modality-specific features and multi-modal fusion features of the former layers. Moreover, locating NPC lesions according to the difference between the left and right sides of the diseased nasopharynx is a valuable way to improve the detection performance of networks. In theory, some networks with transformers can learn the relationship between all patches, including bilaterally symmetrical relationships (Chen et al., 2021; Hatamizadeh et al., 2021; Khan et al., 2021). However, the training of these models requires a large dataset, which is difficult to achieve. In addition, the relationship matrices representing the spatial relation of paired patches are sophisticated and not explainable (Khan et al., 2021). To avoid these issues, the BSP that directly crops T1, T2, DT, and their bilaterally flipped modalities into patches at the same positions is proposed.

## 3. Methods

**FIGURE 1:**
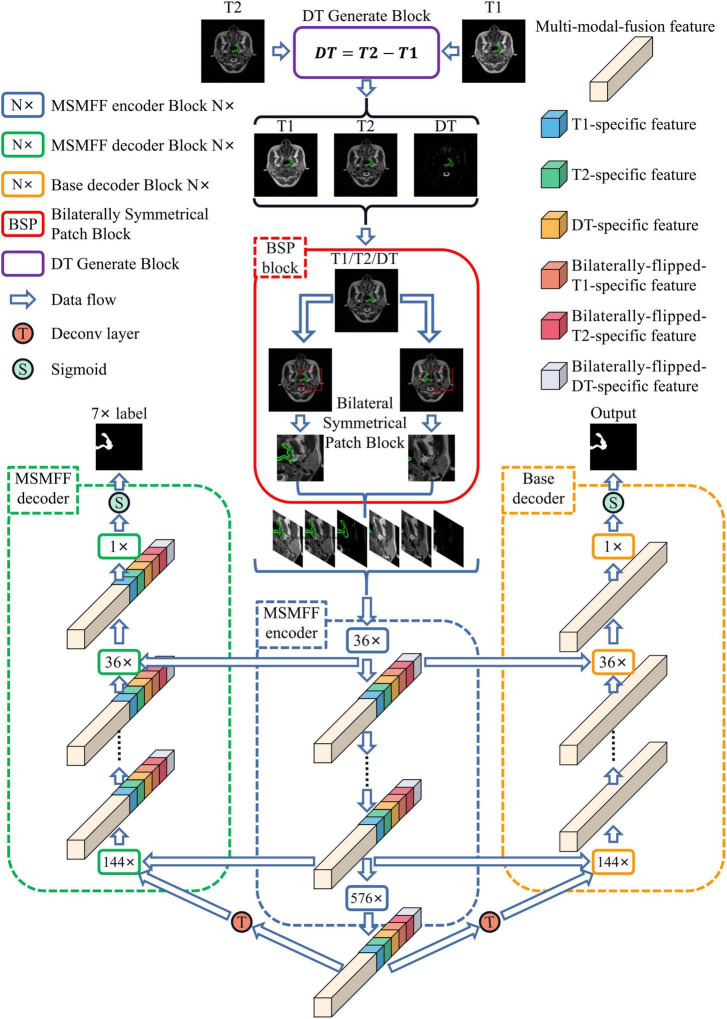
Architecture of BSMM-Net. The BSMM-Net is composed of DT-generated block, bilaterally symmetrical patch block, MSMFF encoder, and base decoder. The MSMFF encoder represents the combination of MSMFF encoder blocks. The MSMFF decoder is used as a deep supervisor in the training step. The inputs of the MSMFF encoder are preprocessed by the bilaterally symmetrical patch block and DT-generated block. In inference, the final segmentation of BSMM-Net is the output of the base decoder, which is composed of the base encoder blocks.

As illustrated in Figure 1, the proposed network, BSMM-Net, which is an end-to-end Unet-like convolutional model, contains the MSMFF encoder and takes patches from T1, T2, DT, and their horizontally flipped modalities as inputs. DT modality, which is the difference between T1 and T2, can intensify the influence of high-water-content tissues, such as the cerebellum and spinal cord, on the network by strengthening their signals. BSP treats the bilaterally flipped images of T1, T2, and DT as independent modalities and crops the flipped and raw images into bilaterally symmetrical patches, which contain the bilaterally symmetrical information of the head that can be utilized to improve the accuracy of tumor detection by the networks. The MSMFF encoder can extract MSMFFs containing the fully complementary information of NPC lesions from modalities. The complementary information in the MSMFF can be aggregated to accurately delineate NPC lesions using the base decoder, which then introduces a convolutional block attention module (CBAM) into its networks to enhance the deep features of NPC lesions. To ensure the validity of MSMFF, the MSMFF decoder is only deployed in the training step to deeply supervise the MSMFF encoder.

### 3.1. Bilaterally symmetrical patch and DT modality

**FIGURE 2:**
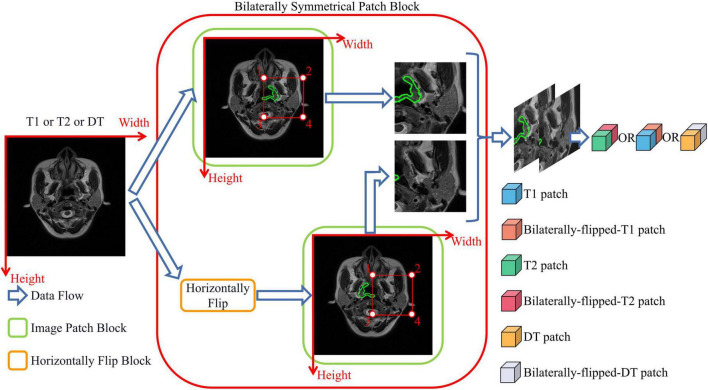
Bilaterally symmetrical patch block. The bilaterally symmetrical patch block crops the images and their horizontally flipped images into patches of the same size. Bilaterally flipped images are treated as an independent modality.

The bilateral symmetry of the head is essential in locating NPC tumors. An image and its horizontally flipped image are simultaneously cropped into bilaterally symmetrical patches to directly and efficiently use the bilateral difference of the head. The cropping process of BSP is presented in Figure 2, where the orange box represents the horizontally flipped block that can flip the left and right sides of the image, and the green box represents the image patch block that can crop the region of the image and the flipped image in the labeled four points into patches. As shown in Figure 2, the structure of normal tissues in the raw image is similar to those in the horizontally flipped image. Meanwhile, the lesion in the green contour breaks this symmetry. T1, T2, and DT modalities are all fed into BSP to fully utilize the information on the bilateral symmetry of the head.

The DT modal image **I**_DT_ is the difference between the T2 modal image **I**_*T2*_ and T1 modal image **I**_*T1*_:


(1) $$I_{\text{DT}}\;\left(x,y\right)=I_{\mathrm{T2}}\;\left(x,y\right)-I_{\mathrm{T1}}$$

where *I*_*T1*_ (*x*,*y*) is the intensity of the T1 image in the position (*x*,*y*), and *I*_*T2*_ (*x*,*y*) is the intensity of the T2 image in (*x*,*y*). The output of the DT-generated block *I*_DTB_ (*x*,*y*) = (*I*_*T1*_ (*x*,*y*),*I*_*T2*_ (*x*,*y*),*I*_DT_ (*x*,*y*)) is a stack of T1, T2, and DT modal images that are cropped into a series of patches by BSP. With the use of BSP and DT-generated block, the proposed network can directly utilize the information on the bilateral symmetry of the head and high-water-content tissues.

### 3.2. Modality-specific and multi-modal fusion features

**FIGURE 3:**
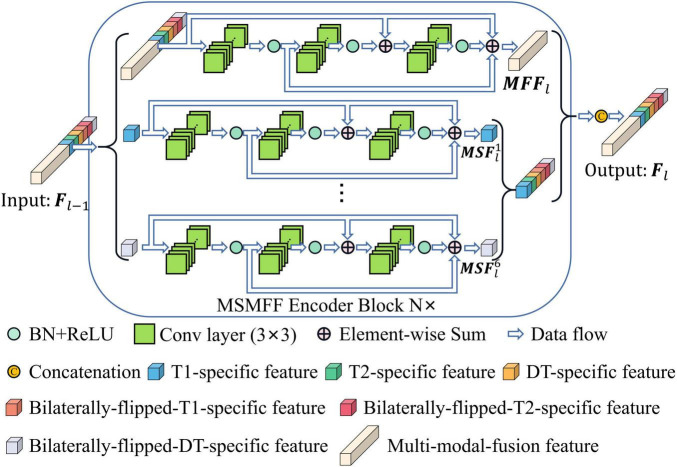
Modality-specific and multi-modal fusion feature encoder block.


All levels of modality-specific and multi-modal fusion features (MSMFFs) are crucial in feature fusion (Lan et al., 2022). A modality-specific feature is only extracted from a specific modality and contains exclusive and complementary information. A multi-modal fusion feature is extracted from the modality-specific features and multi-modal fusion features of previous layers. As shown in Figure 3, the top residual network (He et al., 2016) in the MSMFF encoder blocks extracts multi-modal fusion feature **M***FF*_*l*_ from MSMFF ${\text{F}}_{l-1}\in R^{\frac{H}{2^{l-2}}\times \frac{W}{2^{l-2}}\times \frac{C}{2^{2-l}}}$, where $\text{M}\,F\,F_{l}\in R^{\frac{H}{2^{l-1}}\times \frac{W}{2^{l-1}}\times \frac{C\times 2^{1-l}}{2}}$ is a feature map related to the MSMFFs **F**_*l*−1_ of former layers. Modality-specific features $\text{M}\,S\,F_{l}^{n}\in R^{\frac{H}{2^{l-1}}\times \frac{W}{2^{l-1}}\times \frac{C\times 2^{1-l}}{6\times 2}}$ are, respectively, extracted from the $\text{M}\,S\,F_{l-1}^{n}$ of previous layers by the corresponding residual network in the MSMFF encoder blocks. Here, *n* = 1, 2, 3,4, 5, 6 denote T1 modal patches, T2 modal patches, DT modal patches, bilaterally flipped T1 patches, bilaterally flipped T2 patches, and bilaterally flipped DT patches, respectively, and *l* = 1, 2, 3,4 represent different depths. Given the feature **F**_*l*−1_ of former layer, the output MSMFFs ${\text{F}}_{l}=\left(\text{M}\,F\,F_{l},\text{M}\,S\,F_{l}^{1},\text{M}\,S\,F_{l}^{2},\ldots ,\text{M}\,S\,F_{l}^{6}\right)\in R^{\frac{H}{2^{l-1}}\times \frac{W}{2^{l-1}}\times C\times 2^{1-l}}$ are a concatenation of the multi-modal fusion feature **M***FF*_*l*_ and all modality-specific features $\text{M}\,S\,F_{l}^{n}$. The multi-modal fusion feature **M***FF*_*l*_ can be calculated using the following equation:

$${\text{MFF}}_{l}=R\,e\,s_{l}^{M\,F\,F}\;\left({\text{F}}_{l-1}\right)=B\,a\,s\,e\,B\,l\,o\,c\,k_{l}^{M\,F\,F\,3}$$

(2) $$\left(B\,a\,s\,e\,B\,l\,o\,c\,k_{l}^{M\,F\,F\,2}\;\left(B\,a\,s\,e\,B\,l\,o\,c\,k_{l}^{M\,F\,F\,1}\;\left({\text{F}}_{l-1}\right)\right)+F_{l-1}\right)+B\,a\,s\,e\,B\,l\,o\,c\,k_{l}^{M\,F\,F\,1}\;\left({\text{F}}_{l-1}\right)+F_{l-1}$$

$$B\,a\,s\,e\,B\,l\,o\,c\,k_{l}^{M\,F\,F\,n}\;\left(F\right)=R\,e\,L\,U\;\left(B\,N\;\left({\text{W}}_{l}^{M\,F\,F\,n}*\text{F}+B_{l}^{M\,F\,F\,n}\right)\right)$$

where *BaseBlock*(⋅) is the base block of the proposed network, ${\text{W}}_{l}^{M\,F\,F\,n}$ is the *x*th convolution kernel of the *l*-th multi-modal fusion feature layer, the symbol * represents the convolution operation, and $B_{l}^{M\,F\,F\,n}$ is the bias. *BN*(⋅) and *ReLU*(⋅) are batch normalization and rectified linear units, respectively (Nair and Hinton, 2010; Ioffe and Szegedy, 2015). The modality-specific features $\text{M}\,S\,F_{l}^{n}$ of the MSMFF encoder blocks can be extracted in a similar way by feeding the modality-specific features $\text{M}\,S\,F_{l-1}^{n}$ of previous layers into the corresponding residual network.

**FIGURE 4:**
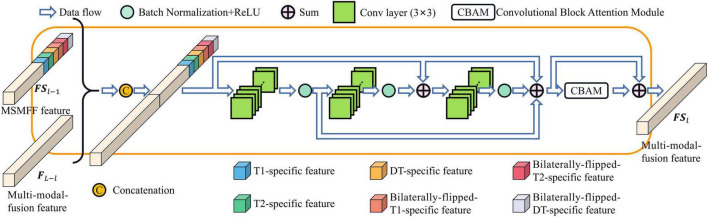
Base decoder block.


As depicted in Figure 1, the MSMFFs are skipped into the base decoder blocks and then fused together to fully utilize the bilateral symmetry of the head and complementary information of modalities in delineating NPC lesions. These base decoder blocks follow the CBAM and residual network for enhancing the difference between the lesions and backgrounds, as illustrated in Figure 4. The MSMFFs **F**_*L*−*l*_ and multi-modal fusion feature **F***S*_*l*−1_ are concatenated and fed into the residual network of a base decoder block. In particular, the residual network applies three 3×3 convolutions to fuse the different level features **F**_*L*−*l*_ and **F***S*_*l*−1_, followed by a CBAM module that enhances spatial features by aggregating global spatial information and channels. Exporting aggregated feature **B***D*_*l*_ from the base decoder is mathematically expressed as Equation 3. *Res*(⋅) is the residual network block-like Equation 2. *CBAM*(⋅) denotes the CBAM, which is a concatenation of the CAM and position attention module (PAM) as described in Equation 4.

(3) $$\text{F}\,S_{l}=C\,B\,A\,M_{l}\;\left(R\,e\,s_{l}^{B\,D}\;\left(\left({\text{F}}_{L-l},\text{F}\,S_{l-1}\right)\right)\right)+R\,e\,s_{l}^{B\,D}\;\left(\left({\text{F}}_{L-l},\text{F}\,S_{l-1}\right)\right)$$

(4) $$C\,B\,A\,M\;\left({\text{F}}_{R\,e\,s}\right)=P\,A\,M\;\left(C\,A\,M\;\left({\text{F}}_{R\,e\,s}\right)\right)$$

$$C\,A\,M\;\left({\text{F}}_{R\,e\,s}\right)=S\,i\,g\,m\,o\,i\,d$$

$$({\text{W}}^{C\,A\,M\,3}*BaseBlock^{C\,A\,M\,2}\;(MaxPool\;(BaseBlock^{C\,A\,M\,1}\;({\text{F}}_{R\,e\,s})))$$

$$+B^{C\,A\,M\,3}+{\text{W}}^{C\,A\,M\,3}*B\,a\,s\,e\,B\,l\,o\,c\,k^{C\,A\,M\,2}$$

$$\left(AvgPool\;(BaseBlock^{C\,A\,M\,1}\;({\text{F}}_{R\,e\,s}))\right)+B^{C\,A\,M\,3})⊗\text{F}{}_{R\,e\,s}$$

$$PAM\;({\text{F}}_{C\,A\,M})=Sigmoid\;(BaseBlock^{P\,A\,M}$$

$$\left(MaxPool\;({\text{F}}_{C\,A\,M}),AvgPool\;({\text{F}}_{C\,A\,M})\right))⊗\text{F}{}_{C\,A\,M}$$

where *BaseBlock*(⋅) is shown in Equation 2. *MaxPool*(⋅) and *AvgPool*(⋅) are the max pooling function and average pooling function, respectively. The two pooling functions compute spatial context descriptors in CAM and downsample along the channel axis in PAM. ⊗ denotes element-wise multiplication. *Sigmoid*(⋅) is a Sigmoid function to generate channel attention and spatial attention. With these functions, the base decoder can aggregate validating features and precisely contour the NPC lesions.

**FIGURE 5:**
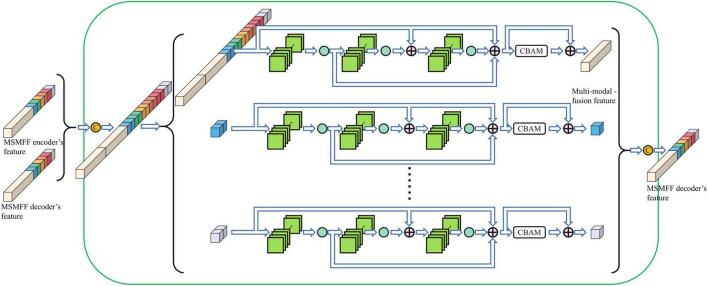
Modality-specific and multi-modal fusion feature decoder block.

Compared with the encoder of Unet, the structure of the MSMFF encoder is more intricate and needs a large number of datasets for training. The limited size of the NPC MRI dataset is not large enough to support the training of the MSMFF encoder; therefore, the MSMFF decoder is proposed as a deep supervisor (Lee et al., 2015) to supervise the MSMFF encoder during training to improve the validity of MSMFFs. As shown in the green dashed frame of Figure 1, the MSMFF decoder is composed of MSMFF decoder blocks, as depicted in Figure 5. Similar to MSMFF encoder blocks, the output of MSMFF decoder block is a concatenation of modality-specific features and multi-modal fusion features generated by base decoder-like networks using the MSMFFs from previous MSMFF decoder block and MSMFF encoder. Supervised by the modality-specific residual network in MSMFF decoder blocks, the modality-specific residual network block in the MSMFF encoder can extract more target-relating modality-specific features than the one only supervised by the base decoder. The multi-modal fusion residual network block in the MSMFF decoder prompts the MSMFF encoder to aggregate more validating complementary information of different modalities than without the MSMFF decoder.

BSMM-Net consists of the abovementioned MSMFF encoder and base decoder and can achieve a good outline sketching performance by fully utilizing the bilateral symmetry of the head and complementary information of modalities. Supervised by the MSMFF decoder, the MSMFF encoder can extract representative MSMFFs from the bilaterally symmetrical patches. The base encoder outputs the final prediction of BSMM-Net by aggregating the MSMFFs.

## 4. Experiments

### 4.1. Data

The BSMM-Net is tested on the NPC MRI dataset from the Chinese Sun Yat-sen University Cancer Center. The dataset contains 7,633 pairs of T1 and T2 modalities from 745 patients who were treated at Sun Yat-sen University Cancer Center from January 2010 to January 2013. The training and testing sets include 670 (7,034 slices) and 75 (599 slices) patients.

All MRIs are scanned by cross-sectional position using 1.5-T or 3.0-T MRI systems with head-neck combined coils. The scanning parameters are as follows: T1 (FSE, axial plane, TR = 540 ms, TE = 11.8 ms) and T2 (FSE, axial plane, TR = 4,000 ms, TE = 99 ms). The resolution of MRIs is different from 320 × 320 to 512 × 512. In particular, 7,633 slices with NPC lesions are chosen before normalization. First, three radiologists individually draw the labels following the same delineation protocol and then review them together and decide on the final labels. Patients are selected according to the following rules: presence of biopsy-proven NPC, undergoing intensity-modulated radiation therapy, and presence of complete imaging and clinical data.

### 4.2. Training details and evaluation metrics

The proposed BSMM-Net is trained, evaluated, and tested using PyTorch 1.9 with the CUDA 11.4 and a single NVIDIA RTX 2080 Ti GPU. Adam optimizer is used for training the network. The betas, weight decay, and learning rate of Adam are, respectively, set to (0.9, 0.9999), 0.001, and 0.0001. The training process is stopped when the loss function on the validation set stops decreasing over 7 epochs to avoid overfitting. A horizontal flip is conducted for all images of the train set for data augmentation. Patches with size 128 × 128 are cropped as inputs that are randomly shuffled before each epoch during training to solve the problem of the variable sizes of images. The input of the multi-modal fusion network of the first MSMFF encoder block is a concatenation of patches of six modalities. Additionally, the input of the modality-specific net is patches of the corresponding modality. In the proposed network, an MSMFF decoder is used to supervise the proposed MSMFF encoder through the auxiliary loss function *L*_*a*_. A principal loss function *L*_*p*_ is also applied to optimize the whole network consisting of MSMFF encoder and base decoder. The auxiliary loss function *L*_*a*_ and principal loss function *L*_*p*_ are the linear combinations of soft dice loss *L*_*DICE*_ and cross-entropy loss *L*_*CE*_, respectively, as shown in Equations 5, 6 (Li and Lee, 1993; Bertels et al., 2020):


$$L_{a}=\alpha_{a}\cdot L_{D\,I\,C\,E}$$

(5) $$\left({\text{GT}}_{7},{\text{Pred}}_{M\,S\,M\,F\,F}\right)+\beta_{a}\cdot L_{C\,E}\;\left({\text{GT}}_{7},{\text{Pred}}_{M\,S\,M\,F\,F}\right)$$

$$L_{p}=\alpha_{p}\cdot L_{D\,I\,C\,E}$$

(6) $$\left(\mathrm{GT},{\text{Pred}}_{b\,a\,s\,e}\right)+\beta_{p}\cdot L_{C\,E}\,\left(\mathrm{GT},{\text{Pred}}_{b\,a\,s\,e}\right)$$

where ***GT*** means the ground truth and is delineated by radiologists. ***GT*_7_** is a stack of seven same-ground truths ***GT***. ***Pred****_*MSMFF*_* is the output of the MSMFF decoder and is only used to supervise the MSMFF encoder during training. *Pred*_*base*_ aggregates multilevel, multi-modal, and bilaterally symmetrical features from the MSMFF encoder and is the output of the base decoder. Hyperparameters *α_*a*_*, *β_*a*_*, *α_*b*_*, and *β_*b*_* are all empirically set to 0.25.

Dice similarity coefficient (DICE), precision (PREC), recall (RECALL), and the intersection of union (IoU) are adopted to evaluate the segmentation performance. These evaluation metrics are calculated using Equations 7–10:


(7) $$D\,I\,C\,E=\frac{2\,N\,U\,M\;\left(\text{G}\,T\;\cap \;{\text{Pred}}_{b\,a\,s\,e}\right)}{N\,U\,M\;\left(\text{G}\,T\right)+N\,U\,M\;\left(\text{P}\,r\,e\,d_{b\,a\,s\,e}\right)}$$

(8) $$P\,R\,E\,C=\frac{N\,U\,M\;\left(\text{G}\,T\;\cap \;{\text{Pred}}_{b\,a\,s\,e}\right)}{N\,U\,M\;\left(\text{P}\,r\,e\,d_{b\,a\,s\,e}\right)}$$

(9) $$R\,E\,C\,A\,L\,L=\frac{N\,U\,M\;\left(\text{G}\,T\;\cap \;{\text{Pred}}_{b\,a\,s\,e}\right)}{N\,U\,M\;\left(\text{G}\,T\right)}$$

(10) $$I\,o\,U=\frac{N\,U\,M\;\left(\text{GT}\;\cap \;{\text{Pred}}_{b\,a\,s\,e}\right)}{N\,U\,M\;\left({\text{Pred}}_{b\,a\,s\,e}\right)+N\,U\,M\;\left({\text{Pred}}_{b\,a\,s\,e}\right)-N\,U\,M\;\left(\text{GT}\;\cap \;{\text{Pred}}_{b\,a\,s\,e}\right)}$$

where *NUM*(⋅) is the number of pixels divided into lesions by segmentation models or radiologists. The mean evaluation metric of global slices is denoted by subscript “S.” The average of the evaluation metrics of all patient cases is a metric for 3D results and is denoted by subscript “V.” For example, *DICE*_*S*_ means the global dice similarity coefficient, and *DICE*_*V*_ represents the case dice similarity coefficient.

### 4.3. Comparison with state-of-the-art

The detailed numerical results of BSMM-Net and compared models including base Unet (Ronneberger et al., 2015), SwinUnet (Cao et al., 2021), TransUnet (Lan et al., 2022), and BASNet (Qin et al., 2019) are summarized in Table 1. For the compared models, the inputs are a concatenation of T1, T2, and DT images. The base Unet, which is the baseline of the proposed method, is an Unet with batch normalization and residual networks. Similar to the proposed BSMM-Net, the feeding inputs are cropped. For the other compared models, the input images are resized according to reference. As shown in Tables 1, 2, the proposed model achieves the results with *DICE*_*S*_, *PREC*_*S*_, *RECALL*_*S*_, *IoU*_*S*_, *DICE*_*V*_, *PREC*_*V*_, *RECALL*_*V*_, and *IoU*_*V*_ of 0.82 ± 0.12, 0.82 ± 0.15, 0.86 ± 0.13, 0.72 ± 0.15, 0.85 ± 0.06, 0.83 ± 0.10, 0.87 ± 0.06, and 0.74 ± 0.08, respectively. The bold value indicates that the metrics of related results are significantly better than those of the compared model that ranked second. Except for precision, the other metrics of the proposed method are significantly better than those of the compared models. The *t*-test results for the proposed method vs. the competing method are shown in Table 3 and prove the significant difference in results between the proposed model and compared models. According to Tables 1, 2, a comparison of the results of base Unet and SwinUnet reveals that the metrics of volumes are not in direct proportion to the metrics of slices. The next best model to DICE and IoU is the TransUnet in terms of slices and volumes.

**TABLE 1:** Comparison of different models in slices (2D).

Method	*DICE*_S_	*PREC*_S_	*RECALL*_S_	*IoU*_S_
Base Unet	0.76 ± 0.20	0.75 ± 0.20	0.81 ± 0.23	0.64 ± 0.20
SwinUnet	0.76 ± 0.17	**0.86** ± 0.16	0.71 ± 0.20	0.63 ± 0.18
TransUnet	0.78 ± 0.15	0.82 ± 0.16	0.78 ± 0.18	0.66 ± 0.17
BASNet	0.66 ± 0.26	0.78 ± 0.27	0.61 ± 0.27	0.53 ± 0.24
BSMM-Net	**0.82** ± 0.12	0.82 ± 0.15	**0.86** ± 0.13	**0.72** ± 0.15

**TABLE 2:** Comparison of different models in volumes (3D).

Method	*DICE*_V_	*PREC*_V_	*RECALL*_V_	*IoU*_V_
Base Unet	0.76 ± 0.20	0.75 ± 0.20	0.82 ± 0.23	0.64 ± 0.20
SwinUnet	0.80 ± 0.07	**0.87** ± 0.09	0.74 ± 0.09	0.67 ± 0.09
TransUnet	0.81 ± 0.07	0.84 ± 0.10	0.80 ± 0.09	0.69 ± 0.08
BASNet	0.72 ± 0.11	0.83 ± 0.11	0.65 ± 0.14	0.57 ± 0.12
BSMM-Net	**0.85** ± 0.06	0.83 ± 0.10	**0.87** ± 0.06	**0.74** ± 0.08

**TABLE 3:** The *t*-test results for the proposed method vs. the competing method.

Paired *t*-test	*P*-value of *DICE*_S_	*P*-value of *PREC*_S_	*P*-value of *RECALL*_S_	*P*-value of *IoU*_S_
BSMM-Net vs. base Unet	††	∼	††	††
BSMM-Net vs. SwinUnet	††	*	††	††
BSMM-Net vs. TransUnet	††	††	††	††
BSMM-Net vs. BASNet	††	†	††	††

^∼^Nonsignificant, *p ≤ 0.05, ^†^*p* ≤ 0.005, ^†⁣†^*p* ≤ 0.001.

**FIGURE 6:**
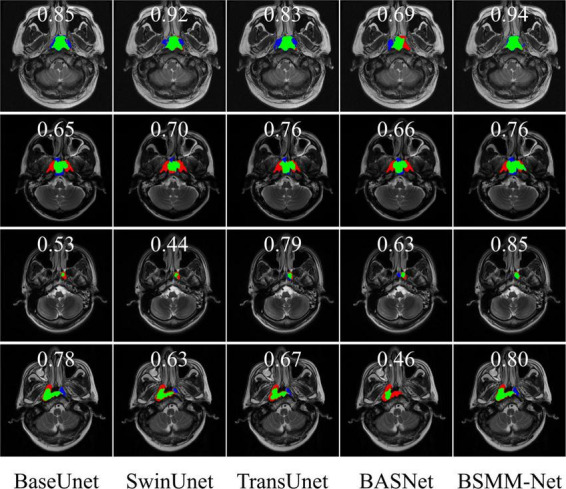
Visualization of segmentation masks. The green regions are the NPC lesion regions correctly segmented by the models. The blue regions are the false positive regions erroneously divided into targets by models. The red regions are the false negative regions and the regions of NPC lesions missed by the models.

The representative visual segmentation examples are presented in Figure 6. The green regions are the true positive region, which is the ground truth and correctly contoured by models. The blue regions represent the false positive region, which is the background and delineated into NPC lesions by the models. The red regions mean the false negative region, which is the ground truth and is overlooked by the models in predicting the NPC lesions. The corresponding *DICE*_*S*_ of each visualized image are shown on top of the image. From the third and fourth rows of Figure 6, the BSMM-Net can achieve better segmentation than other models from the images whose NPC lesions have bad left and right symmetry. A comparison between the rightmost columns of Figure 6 with other columns reveals that the results of BSMM-Net are more similar to those of the manual segmentation by radiologists.

### 4.4. Ablation study

The validity of BSP, a decoder with CBAM and MSMFF encoder, is validated in this subsection, and the results are illustrated in Table 4. The notation “√” means the utilization of the modules. The bold value indicates that the metrics of related results are best. The base Unet, which is a simple Unet-like network, is the BSMM-Net without CBAM, BSP, and modality-specific residual networks of MSMFF encoder. When BSP is applied or the decoder with CBAM is deployed, all the evaluation metrics are substantially improved. However, when BSP and CBAM are applied concurrently, the RECALL is improved and the PREC drops. According to the fifth row of Table 4, the DICE, PREC, and IoU of model with the MSMFF encoder can also achieve remarkable improvement. The Table 5 shows the *t*-test results for the base Unet vs. enhancements. According to the Table 5, except the difference of *RECALL*_*S*_ between base Unet and base Unet with BSP module is nonsignificant, the other *p*-values are significant.

**TABLE 4:** Nasopharyngeal carcinoma segmentation results of ablation experiments.

Method				*DICE*_S_	*PREC*_S_	*RECALL*_S_	*IoU*_S_
Base Unet	BSP	CBAM decoder	MSMFF encoder				
√				0.76 ± 0.20	0.75 ± 0.20	0.81 ± 0.23	0.64 ± 0.20
√	√			0.81 ± 0.14	0.82 ± 0.16	0.83 ± 0.16	0.70 ± 0.17
√		√		0.81 ± 0.14	0.82 ± 0.16	0.84 ± 0.16	0.70 ± 0.17
√	√	√		0.81 ± 0.14	0.79 ± 0.15	**0.87 ± 0.16**	0.70 ± 0.16
√	√	√	√	**0.82 ± 0.12**	0.82 ± 0.15	0.86 ± 0.13	**0.72 ± 0.15**

“√” marks represent utilized modules.

**TABLE 5:** Hypothesis validation results of the base Unet vs. enhancements.

Paired *t*-test	*P*-value of *DICE*_S_	*P*-value of *PREC*_S_	*P*-value of *RECALL*_S_	*P*-value of *IoU*_S_
Base Unet vs. base Unet + BSP	††	††	∼	††
Base Unet vs. base Unet + CBAM decoder	††	††	*	††
Base Unet vs. base Unet + BSP + CBAM decoder	††	††	††	††
Base Unet vs. BSMM-Net	††	††	††	††

^∼^Nonsignificant, *p ≤ 0.05, ^†⁣†^*p* ≤ 0.001.

## 5. Discussion

This study proposes a neural network to delineate NPC in MRI images. Results show that BSMM-Net significantly outperforms state-of-the-art techniques in terms of DICE, RECALL, and IoU metrics. This improvement can be attributed to the use of the bilateral symmetry of the head and the complementary information aggregated from MSMFFs. As listed in Tables 1, 2, the PREC of SwinUnet is higher and the other metrics are lower than those of TransUnet, which also utilizes transformers. This finding indicates that the SwinUnet missed more true positive regions than TransUnet because the former utilizes the sliding window to relieve the computation of transformers. Some long-range or symmetrical relationships, which are important for locating NPC lesions, can be elided by the sliding windows of SwinUnet. The difference in performance between SwinUnet and TransUnet proves that the long-range spatial relationship including the bilateral symmetry of the head is a key for detecting NPC lesions. Although resnet34 (Li et al., 2021) used as an encoder by BASNet is pre-trained in the ImageNet datasets, the dataset is not large enough to train a large number of parameters of BASNet. Therefore, the BASNet achieved the worst performance in the experiments. According to Tables 1, 2, the precision of the BSMM-Net is lower than that of the SwinUnet and TransUnet. The lower precision of the proposed model means more backgrounds are segmented into NPC lesions by the BSMM-Net than by the SwinUnet and TransUnet. Since the proposed model is trained through cropped images, the global information of the whole images is lost and the BSMM-Net predicts more false positive regions. Compared with the false positive regions caused by the proposed model, the far more true positive regions by the proposed model than the compared models are more valuable in other metrics and more useful in the practical clinic. Compared with the TransUnet, BSMM-Net can more directly utilize the information on the bilateral symmetry of the head through the bilaterally symmetrical patches. The MSMFF encoder can aggregate more representative complementary information than the compared models by utilizing the MSMFFs. Hence, BSMM-Net is feasibly deployed to delineate NPC tumors in practical applications.

In the ablation study, BSP shows significant improvements attributed to the bilateral symmetry of the head. The decoder with CBAM also achieves similar improvement to BSP by re-weighing the features of pixels through the attention generated from the global and channel information. However, the DICE and IoU of the model utilizing BSP and decoder with CBAM have no difference from those of the models only using BSP or CBAM. In addition, the RECALL and PREC of the model with BSP and CBAM improve and decrease, respectively. BSP and CBAM allow the model to correctly segment some NPC regions that are missed by each other, thus improving the RECALL of the model with both these blocks. However, the poor PREC means that the backgrounds segmented into targets by the models with BSP and models with CBAM decoder are also divided into targets by the model with BSP and CBAM. As listed in Table 4, the MSMFF encoder can further improve the DICE and IoU by utilizing MSMFFs. This finding proves that the proposed MSMFF encoder can aggregate more complementary information from different modalities than directly feeding the modalities. The ablation study and comparison with state-of-the-art models demonstrate that the bilateral symmetry of the head and complementary information aggregated from the MSMFF encoder can improve the precision of NPC segmentation. In general, the proposed method can assist physicians to delineate the primary tumors of NPC on multi-modal MRIs.

Even though the proposed method attains great improvement in the NPC delineation task, some limitations remain to be solved in future studies. (1) In this study, the number of datasets is insufficient at present. In the follow-up study, additional MRI images will be acquired to improve the robustness and generalization of the proposed method. (2) The proposed model is experimented with in 2D form and, thus, may overlook the effect of 3D spatial information of the head. The future study will utilize the 3D spatial information to achieve improvements. (3) The dataset used in this article expels the images obtained when the patient’s position conspicuously tilts to one side. If images with asymmetrical positions can be rotated to ensure the bilaterally symmetrical patient position, then the proposed method can achieve further improvement. Therefore, the proposed method can further improve the segmentation of NPC for future use.

## 6. Conclusion

In this study, BSMM-Net is proposed to precisely delineate NPC in multi-modality MRI of the head. The baseline of the proposed model is an Unet-like network, which can utilize long-range skipping to fuse high-level and low-level features. The raw image and its horizontally flipped one are cropped into bilaterally symmetrical patches, which are then directly fed into the proposed network to simulate radiologists locating NPC lesions according to the difference between the left and right sides of the head. For the full utilization of the complementary information between modalities, the MSMFF encoder is designed to extract MSMFFs. An MSMFF decoder that is structurally similar to the MSMFF encoder is used as a deep supervisor during training. The experiments demonstrate the effectiveness of the proposed method on a clinical dataset. The mean dice similarity scores of the proposed model are better than the baseline and those of SwinUnet, TransUnet, and BASNet at 0.82 ± 0.12 and 0.86 ± 0.06 in slices and cases. The superiority of BSMM-Net can mostly be attributed to the complementary advantages of the proposed methods.

## Data availability statement

For ethical considerations and protecting the privacy of patients, the data set used in this manuscript is not publicly available according to the regulations of Sun Yat-sen University Cancer Center. Although the data set is not open-access, other researchers can request the data set and codes through the corresponding authors and signing the use agreements for verifying this manuscript.

## Ethics statement

The studies involving human participants were reviewed and approved by Sun Yat-sen University Cancer Center. The Ethics Committee waived the requirement of written informed consent for participation.

## Author contributions

HZ: conceptualization, methodology, software, investigation, formal analysis, and writing—original draft. HL: data curation and writing—original draft. SC: visualization and investigation. SY: software and validation. GR: resources and supervision. LL: resources, supervision, and writing—review and editing. HC: conceptualization, funding acquisition, resources, supervision, and writing—review and editing. All authors contributed to the article and approved the submitted version.

## References

[B1] Bai X. Hu Y. Gong G. Yin Y. Xia Y. (2021). A deep learning approach to segmentation of nasopharyngeal carcinoma using computed tomography. *Biomed. Signal Proc. Control.* 64:102246. 10.1016/j.bspc.2020.102246

[B2] Bertels J. Robben D. Vandermeulen D. Suetens P. (2020). “Optimization with soft dice can lead to a volumetric bias,” in *Brainlesion: Glioma, Multiple Sclerosis, Stroke and Traumatic Brain Injuries*, eds Crimi A. Bakas S. (Cham: Springer International Publishing), 89–97. 10.1007/978-3-030-46640-4_9

[B3] Cai M. Wang J. Yang Q. Guo Y. Zhang Z. Ying H. (2021). Combining images and t-staging information to improve the automatic segmentation of nasopharyngeal carcinoma tumors in MR images. *IEEE Access* 9:21323–21331. 10.1109/ACCESS.2021.3056130

[B4] Cao H. Wang Y. Chen J. Jiang D. Zhang X. Tian Q. (2021). Swin-Unet: unet-like pure transformer for medical image segmentation. *ArXiv[Preprint]* Available online at: http://arxiv.org/abs/2105.05537 (accessed October 29, 2021).

[B5] Chanapai W. Bhongmakapat T. Tuntiyatorn L. Ritthipravat P. (2012). Nasopharyngeal carcinoma segmentation using a region growing technique. *Int. J. CARS* 7 413–422. 10.1007/s11548-011-0629-6 21671094

[B6] Chen H. Qi Y. Yin Y. Li T. Liu X. Li X. (2020). MMFNet: a multi-modality MRI fusion network for segmentation of nasopharyngeal carcinoma. *Neurocomputing* 394 27–40. 10.1016/j.neucom.2020.02.002

[B7] Chen J. Lu Y. Yu Q. Luo X. Adeli E. Wang Y. (2021). TransUNet: transformers make strong encoders for medical image segmentation. *ArXiv[Preprint]* Available online at: http://arxiv.org/abs/2102.04306 (accessed November 23, 2021).

[B8] Dhamija T. Gupta A. Gupta S. Anjum, Katarya R. Singh G. (2022). Semantic segmentation in medical images through transfused convolution and transformer networks. *Appl. Intell.* 10.1007/s10489-022-03642-w 35498554 PMC9035506

[B9] Guo F. Shi C. Li X. Wu X. Zhou J. Lv J. (2020). Image segmentation of nasopharyngeal carcinoma using 3D CNN with long-range skip connection and multi-scale feature pyramid. *Soft Comput.* 24 12671–12680. 10.1007/s00500-020-04708-y

[B10] Hatamizadeh A. Tang Y. Nath V. Yang D. Myronenko A. Landman B. (2021). UNETR: transformers for 3D medical image segmentation. *ArXiv[Preprint]* Available online at: http://arxiv.org/abs/2103.10504 (accessed January 18, 2022). 10.1371/journal.pone.0275033 36223330 PMC9555672

[B11] He K. Zhang X. Ren S. Sun J. (2016). Identity mappings in deep residual networks. *ArXiv[Preprint]* Available online at: http://arxiv.org/abs/1603.05027 (accessed October 10, 2022).

[B12] Hu L. Li J. Peng X. Xiao J. Zhan B. Zu C. (2022). Semi-supervised NPC segmentation with uncertainty and attention guided consistency. *Knowledge Based Syst.* 239:108021. 10.1016/j.knosys.2021.108021

[B13] Huang K.-W. Zhao Z.-Y. Gong Q. Zha J. Chen L. Yang R. (2015). “Nasopharyngeal carcinoma segmentation via HMRF-EM with maximum entropy,” in *Proceedings of the 2015 37th Annual International Conference of the IEEE Engineering in Medicine and Biology Society (EMBC)*, (Milan: IEEE), 2968–2972. 10.1109/EMBC.2015.7319015 26736915

[B14] Huang W. Chan K. L. Zhou J. (2013). Region-based nasopharyngeal carcinoma lesion segmentation from mri using clustering- and classification-based methods with learning. *J. Digit. Imaging* 26 472–482. 10.1007/s10278-012-9520-4 22854973 PMC3649041

[B15] Ioffe S. Szegedy C. (2015). “Batch normalization: accelerating deep network training by reducing internal covariate shift,” in *Proceedings of the 32nd International Conference on Machine Learning*, eds Bach F. Blei D. (Lille: PMLR), 448–456. 10.1007/s11390-020-0679-8

[B16] Ke L. Deng Y. Xia W. Qiang M. Chen X. Liu K. (2020). Development of a self-constrained 3D DenseNet model in automatic detection and segmentation of nasopharyngeal carcinoma using magnetic resonance images. *Oral Oncol.* 110:104862. 10.1016/j.oraloncology.2020.104862 32615440

[B17] Khan S. Naseer M. Hayat M. Zamir S. W. Khan F. S. Shah M. (2021). Transformers in vision: a survey. *ArXiv[Preprint]* Available online at: http://arxiv.org/abs/2101.01169 (accessed November 5, 2021). 35893083

[B18] Lan X. Gu X. Gu X. (2022). MMNet: multi-modal multi-stage network for RGB-T image semantic segmentation. *Appl. Intell.* 52 5817–5829. 10.1007/s10489-021-02687-7

[B19] Lee C.-Y. Xie S. Gallagher P. Zhang Z. Tu Z. (2015). “Deeply-supervised nets,” in *Proceedings of the Eighteenth International Conference on Artificial Intelligence and Statistics*, eds Lebanon G. Vishwanathan S. V. N. (San Diego, CA: PMLR), 562–570. 10.3390/s19092009

[B20] Li C. H. Lee C. K. (1993). Minimum cross entropy thresholding. *Pattern Recognit.* 26 617–625. 10.1016/0031-3203(93)90115-D

[B21] Li Q. Xu Y. Chen Z. Liu D. Feng S.-T. Law M. (2018). Tumor segmentation in contrast-enhanced magnetic resonance imaging for nasopharyngeal carcinoma: deep learning with convolutional neural network. *BioMed. Res. Int.* 2018 1–7. 10.1155/2018/9128527 30417017 PMC6207874

[B22] Li X. Tang M. Guo F. Li Y. Cao K. Song Q. (2021). DDNet: 3D densely connected convolutional networks with feature pyramids for nasopharyngeal carcinoma segmentation. *IET Image Process.* 16 39–48. 10.1049/ipr2.12248 35526269

[B23] Li Y. Dan T. Li H. Chen J. Peng H. Liu L. (2022). NPCNet: jointly segment primary nasopharyngeal carcinoma tumors and metastatic lymph nodes in MR images. *IEEE Trans. Med. Imaging* 41 1639–1650. 10.1109/TMI.2022.3144274 35041597

[B24] Lin L. Dou Q. Jin Y.-M. Zhou G.-Q. Tang Y.-Q. Chen W.-L. (2019). Deep learning for automated contouring of primary tumor volumes by MRI for nasopharyngeal carcinoma. *Radiology* 291 677–686. 10.1148/radiol.2019182012 30912722

[B25] Luo X. Liao W. Chen J. Song T. Chen Y. Zhang S. (2021). “Efficient semi-supervised gross target volume of nasopharyngeal carcinoma segmentation via uncertainty rectified pyramid consistency,” in *Medical Image Computing and Computer Assisted Intervention – MICCAI 2021*, eds de Bruijne M. Cattin P. C. Cotin S. Padoy N. Speidel S. Zheng Y. (Cham: Springer International Publishing), 318–329.

[B26] Men K. Chen X. Zhang Y. Zhang T. Dai J. Yi J. (2017). Deep deconvolutional neural network for target segmentation of nasopharyngeal cancer in planning computed tomography images. *Front. Oncol.* 7:315. 10.3389/fonc.2017.00315 29376025 PMC5770734

[B27] Nair V. Hinton G. E. (2010). “Rectified linear units improve restricted boltzmann machines,” in *Proceedings of the 27th International Conference on International Conference on Machine Learning*, (Madison, WI: Omnipress), 807–814.

[B28] Qin X. Zhang Z. Huang C. Gao C. Dehghan M. Jagersand M. (2019). “BASNet: boundary-aware salient object detection,” in *Proceedings of the 2019 IEEE/CVF Conference on Computer Vision and Pattern Recognition (CVPR)*, (Long Beach, CA: IEEE), 7471–7481. 10.1109/CVPR.2019.00766

[B29] Ronneberger O. Fischer P. Brox T. (2015). “U-Net: convolutional networks for biomedical image segmentation,” in *Medical Image Computing and Computer-Assisted Intervention – MICCAI*, Vol. 2015 eds Navab N. Hornegger J. Wells W. M. Frangi A. F. (Cham: Springer International Publishing), 234–241.

[B30] Tang P. Zu C. Hong M. Yan R. Peng X. Xiao J. (2021). DA-DSUnet: dual attention-based Dense SU-net for automatic head-and-neck tumor segmentation in MRI images. *Neurocomputing* 435 103–113. 10.1016/j.neucom.2020.12.085

[B31] Tatanun C. Ritthipravat P. Bhongmakapat T. Tuntiyatorn L. (2010). “Automatic segmentation of nasopharyngeal carcinoma from CT images: region growing based technique,” in *Proceedings of the 2010 2nd International Conference on Signal Processing Systems*, (Dalian: IEEE), V2-537–V2-541. 10.1109/ICSPS.2010.5555663

[B32] Wong L. M. Ai Q. Y. H. Poon D. M. C. Tong M. Ma B. B. Y. Hui E. P. (2021). A convolutional neural network combined with positional and textural attention for the fully automatic delineation of primary nasopharyngeal carcinoma on non-contrast-enhanced MRI. *Quant. Imaging Med. Surg.* 11 3932–3944. 10.21037/qims-21-196 34476179 PMC8339644

[B33] Xue X. Qin N. Hao X. Shi J. Wu A. An H. (2020). Sequential and iterative auto-segmentation of high-risk clinical target volume for radiotherapy of nasopharyngeal carcinoma in planning CT images. *Front. Oncol.* 10:1134. 10.3389/fonc.2020.01134 32793483 PMC7390915

[B34] Zhao L. Lu Z. Jiang J. Zhou Y. Wu Y. Feng Q. (2019). Automatic nasopharyngeal carcinoma segmentation using fully convolutional networks with auxiliary paths on dual-modality PET-CT Images. *J. Digit. Imaging* 32 462–470. 10.1007/s10278-018-00173-0 30719587 PMC6499852

[B35] Zhou J. Chan K. L. Xu P. Chong V. F. H. (2006). “Nasopharyngeal carcinoma lesion segmentation from MR images by support vector machine,” in *Proceedings of the 3rd IEEE International Symposium on Biomedical Imaging: Macro to Nano, 2006*, (Arlington, VI: IEEE), 1364–1367. 10.1109/ISBI.2006.1625180

[B36] Zhu H. Weibel J.-B. Lu S. (2016). “Discriminative multi-modal feature fusion for RGBD indoor scene recognition,” in *Proceedings of the 2016 IEEE Conference on Computer Vision and Pattern Recognition (CVPR)*, (Las Vegas, NV: IEEE), 2969–2976. 10.1109/CVPR.2016.324

[B37] Zhu X. Wu Y. Hu H. Zhuang X. Yao J. Ou D. (2022). Medical lesion segmentation by combining multimodal images with modality weighted UNet. *Med. Phys.* 49 3692–3704. 10.1002/mp.15610 35312077

